# Microalgae show a range of responses to exometabolites of foreign species

**DOI:** 10.1016/j.algal.2021.102627

**Published:** 2022-03

**Authors:** Natalia G. Apostolopoulou, Evangelia Smeti, Marta Lamorgese, Ioanna Varkitzi, Phillip Whitfield, Clement Regnault, Sofie Spatharis

**Affiliations:** a Department of Ecology and Systematics, National and Kapodistrian University of Athens, 10679, Greece; b School of Life Sciences, University of Glasgow, G12 8QQ, UK; c Institute of Marine Biological Resources and Inland Waters, HCMR Hellenic Centre for Marine Research, PO Box 713, Anavyssos 19013, Greece; d Institute of Oceanography, HCMR Hellenic Centre for Marine Research, PO Box 713, Anavyssos 19013, Greece; e Glasgow Polyomics, University of Glasgow, G61 1BD, UK; f Institute of Biodiversity, Animal Health & Comparative Medicine, University of Glasgow, G12 8QQ, UK

**Keywords:** Microalgae, Exometabolites, Intracellular phosphorus, Luxurious phosphorus uptake, L-histidinal, Tiliacorine

## Abstract

Studies on microalgae interspecific interactions have so far focused either on nutrient competition or allelopathic effects due to excreted substances from Harmful Algal Bloom (HAB) species. Evidence from plants, bacteria and specific microalgae groups, point to a range of responses mediated by sensing or direct chemical impact of exometabolites from foreign species. Such processes remain under-investigated, especially in non-HAB microalgae, despite the importance of such knowledge in ecology and industrial applications. Here, we study the directional effect of exometabolites of 4 “foreign” species *Heterosigma akashiwo*, *Phaeocystis* sp., *Tetraselmis* sp. and *Thalassiosira* sp. to each of three “target” species across a total of 12 treatments. We disentangle these effects from nutrient competition by adding cell free medium of each “foreign” species into our treatment cultures. We measured the biomass response, to the foreign exometabolites, as cell number and photosynthetic biomass (Chla), whereas nutrient use was measured as residual phosphorus (PO_4_) and intracellular phosphorus (P). Exometabolites from filtrate of foreign species were putatively annotated by untargeted metabolomics analysis and were discussed in association to observed responses of target species. Among others, these metabolites included L-histidinal, Tiliacorine and dimethylsulfoniopropionate (DMSP). Our findings show that species show a range of responses with the most common being biomass suppression, and less frequent biomass enhancement and intracellular P storage. Filtrate from the green microalgae *Tetraselmis* caused the most pronounced negative effects suggesting that non-HAB species can also cause negative chemical interference. A candidate metabolite inducing this response is L-histidinal which was measured in high abundance uniquely in *Tetraselmis* and its L-histidine form derived from bacteria was previously confirmed as a microalgal algicidal. *H. akashiwo* also induced biomass suppression on other microalgae and a candidate metabolite for this response is Tiliacorine, a plant-derived alkaloid with confirmed cytotoxic activity.

## Highlights

• We show that microalgae demonstrate a range of responses to foreign exometabolites.
• Biomass suppression caused by *Tetraselmis* was linked to L-histidinal.
• Biomass enhancement was only observed in *Heterosigma akashiwo*.
• Luxurious P uptake can also occur as a response to foreign species exometabolites.
• Tiliacorine is reported for the first time from the mixotroph *H. akashiwo*.

## Graphical abstract

**Figure:**
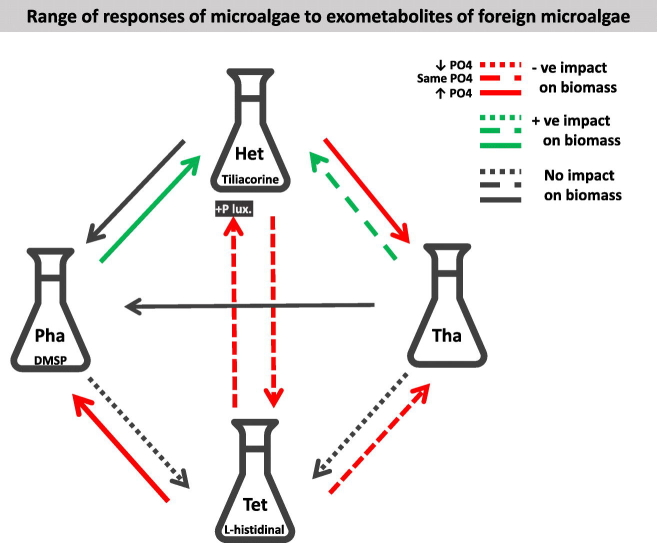

## 1 Introduction

Competition for nutrients is the most well-known process intrinsic to microalgae assemblages leading to predictable composition under specific environmental conditions [1]. On the other hand, chemical interference via metabolites excreted from competitor microalgae (exometabolites) is less well understood. Studies on chemical interference between microalgae have focused on effects of Harmful Algal Bloom (HAB) species recording mostly negative but also positive responses on physiology and growth, aka allelopathy [2]. Evidence from other microbial groups such as bacteria have shown that foreign exometabolites can be sensed by target species and lead to more complex responses including both aggressive and defensive strategies [3]. To advance research in the field it is essential to explore a broader range of responses in microalgae, by expanding experimental settings beyond the effects of single species to crossed designs using HAB and non-HAB species of different taxonomic groups. It is also essential that such investigations, tease apart effects of nutrient competition that are inevitably masking chemical interference in co-cultures. Combining such information with comparative metabolomics of focal species, can shed light into processes sustaining microalgae co-existence, biomass dynamics and nutrient use applicable in field settings and industrial production systems.

The bulk of research on microalgal chemical interference has focused on physiological aspects of cell viability such as growth, biomass and respiration [2]. The main assumption is that compounds excreted from toxin producing species (aka allelochemicals) would impede the growth process and cell viability of the target species via direct chemical effects such as algicidal action. This raises the question on whether microalgae are able to respond to foreign species, via processes other than physiological impairment, as a result of sensing the exometabolites of their competitor. Competition sensing has been reported in bacteria, where toxins released induce responses to competitor species [3]. However, more complex interactions (i.e. quorum quenching) have also been reported whereby the release of toxins by competitor species interferes with substances used for communication between conspecifics of other species (known as quorum sensing signals) [4]. Defensive strategies are also known to occur in bacteria as a response to chemical cues, including damage repair or changes in gene regulation [5]. For example, nutrient starvation leads to transcriptional switching from proliferation-related genes to maintenance-related genes in *Escherichia coli* [6]. In microalgae, a maintenance-related mechanism is luxurious phosphorus (P) uptake, a well-documented process whereby cells adapt to nutrient fluctuations by maximizing their intracellular P storage [7], [8]. Luxurious P uptake might either lead other species, whose P uptake is slower, to starvation, or storing more P than required for growth for supporting other potentially defensive functions [8]. It is thus plausible, that under stress conditions induced by the sensing of competitors, a species shifts its strategy from growth to nutrient storage to gain a competitive advantage over the long term. This process has not yet been explicitly investigated as a response to foreign species exometabolites and could provide the first basis of understanding sensing or direct chemical impacts between microalgae when investigated alongside biomass responses and accompanying information on foreign exometabolites.

Investigations of chemical interference between microalgae species have focused on effects of toxin-producing, Harmful Algal Bloom (HAB) species on other toxic and non-toxic microalgae [2]. The bulk of these studies has reported growth inhibition on target microalgae linked to cell lysis, shifts in respiration, protein synthesis and gene expression [9], [10], [11], [12], [13], [14], [15], [16], [17]. However, positive allelopathy has also been reported by a limited number of studies [15], [18], [19], [20]. This suggests that microalgae do not show a consistent response to exometabolites of foreign microalgae even if the latter are toxin-producing species known to cause adverse effects to higher trophic levels during HABs. Such differences in response directionality are hindering our understanding of community assembly and prediction of phytoplankton assemblage composition while increasing the uncertainty of co-cultivation outcomes when aiming to maximize industrial microalgae production. It is thus critical to understand whether microalgae can respond to exometabolites of non-HAB foreign microalgae and whether such effects extend beyond biomass and physiological changes to defensive strategies such as nutrient use.

In regards to non-HAB species, chemical effects have reported negative allelopathy [21], [22], [31], [32], [23], [24], [25], [26], [27], [28], [29], [30]. However, chemical interference is often tested in experimental designs involving co-cultured species sharing common resources. This poses a challenge in disentangling the effects of chemical interference from competition for nutrients. According to theory, a co-culture of two co-occurring species growing under a constant resource supply in continuous cultures, will end up being exclusively occupied by the species that is more competitive for the limiting resource [1]. It is thus plausible that negative effects to co-cultured target microalgae are not due to chemical interference but rather to the higher competitive ability of the focal species for the limiting resource [32]. Although it is possible to isolate the effect of allelopathy from resource competition by using either culture filtrate or cellular lipophilic extract of the focal species [24], [33], [34], [35], it is impossible to isolate the effect of resource competition from that of chemical interference in co-cultures [32], [36]. This poses the necessity to investigate chemical interference in isolation, while carefully controlling for nutrient concentrations, as an essential step towards understanding how both processes act antagonistically or synergistically to sustain species co-existence and biomass.

To obtain further insights into the mechanisms governing the responses of microalgae to competitor species exometabolites, it is necessary to characterize the exometabolites excreted from a target species. Metabolomics recognizes that alterations in cell function are perhaps more evident at the level of small molecule metabolism and offers a powerful biochemical approach for revealing molecular phenotypes [37], [38]. In the context of this study, secondary metabolites are a key focus, i.e. substances that are not essential for algal growth and that mostly participate in the defensive and protective mechanisms of the cell. Many components in the algal exometabolome can cause interspecific allelopathic effects [39] and the potential of metabolomics to investigate chemical interactions has been already highlighted by different studies [20], [40].

The aim of this study was thus to investigate the response of each of 4 target microalgal species of different taxonomic groups to the presence of exometabolites from each of the other 3 species. The exposure to the exometabolome was performed in the absence of resource competition, by using a nutrient-enriched cell free filtrate, and we quantified responses related to biomass yield (photosynthetic activity and cell number) and phosphorus use (intracellular P storage and residual P in the medium). These responses were linked to specific exometabolites following comparative analysis of the metabolic profile of monoculture filtrate from each species. Evidence that exometabolites of foreign species can lead to multiple responses regarding biomass production and nutrient use could lead to a shift in the way that we predict microalgae dynamics and composition outcomes and can influence decisions on species co-cultivations in industrial biomass production.

## 2 Material and methods

### 2.1 Experimental design & procedure

**Fig. 1:**
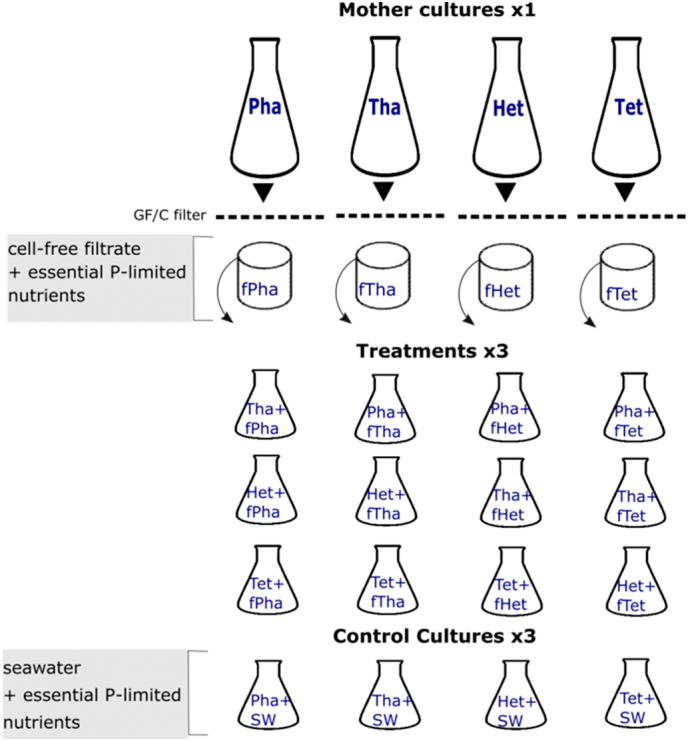
Experimental design testing for the effect of filtrate with exometabolites from four “foreign” microalgae on the biomass and nutrient use by “target” microalgae representing four taxonomic groups: *Heterosigma akashiwo* (Ochrophyta), *Phaeocystis* sp. (Haptophyta), *Tetraselmis* sp. (Chlorophyta) and *Thalassiosira* sp. (Bacillariophyta). The experiment consisted of the treatments whereby triplicate monocultures of each species were treated with filtrate from each “foreign” species MC (fspecies) and controls whereby we added plain artificial seawater (SW) in triplicate monocultures of each species. The total sample size was thus 48 monocultures: 4 “target” species × (3 “foreign” species treatments + 1 control) × 3 replicates.

To address the research questions, we used four HAB and non-HAB marine phytoplankton species representing different taxonomic groups: *Heterosigma akashiwo* (Ochrophyta) and *Phaeocystis* sp. (Haptophyta), both known to form HABs [41], [42], [43], *Tetraselmis* sp. (Chlorophyta) and *Thalassiosira* sp. (Bacillariophyta). A high volume (2 L) mother culture (MC) of each species was used to obtain the cell-free filtrate, by filtration through GF/C glass microfiber filters, which was then added in our treatment cultures. For each species, there were three treatments where MC filtrate of each “foreign” species was added and a control treatment where plain seawater was added (Fig. 1). Each treatment and control comprised of three replicate cultures (200 mL/flask). Thus, the experimental design comprised of 4 species × (3 treatments+1control) × 3 replicates = 48 cultures.

We wanted to see the effect of the addition of foreign species' filtrate on the biomass and nutrient use of the target species at stationary phase, thus aiming at testing for effects at the population level. Two filtrate additions were carried out to intensify any effect of allelochemicals on the treatment cultures mimicking conditions also encountered in the field where species are exposed in a more continuous manner to exometabolites of foreign species. The aim was to test the effects and responses of microalgae when different species interact via their exometabolome, rather than to quantify the exometabolite dose concentrations that would cause the observed effects. Additions occurred when MCs of the four species had reached stationary phase because at this stage allelochemicals from microalgae are in higher concentrations [35], [44]. At this stage, susceptibility of target species to allelochemicals was also expected to be higher than the control since they were under higher stress due to nutrient limitation [45]. The first filtrate addition occurred when treatment and control cultures were at the end of the exponential phase and the second occurred two days later, when cultures had reached stationary phase. Based on pilot data, the growth rate of each species was calculated (see supplementary material “methods”) so that all species' cultures would reach stationary phase simultaneously. Samples were collected at two time points. The first sampling occurred prior to the first filtrate addition, to record the initial biomass of the cultures before any effects of exometabolites took place. These measurements were included in our statistical models to account for differences in initial conditions. The second sampling took place two days after the second addition to allow sufficient time for the populations to react to foreign exometabolites.

In each of the two filtrate addition time points we removed 30% of the volume (i.e. 60 mL) from each of the 48 cultures and replaced it with the same volume of MC filtrate for our treatment cultures or plain seawater for our controls. At these time points residual P concentrations were very low and prior to the addition, essential nutrients were added into treatments and control cultures (i.e. F/2 with P-limitation) to minimize any effects of residual nutrients in the MC on the growth of treatment cultures.

### 2.2 Experimental conditions

Phytoplankton cultures of *Phaeocystis* sp. and *Thalassiosira* sp. were obtained from the algal collection of the Hellenic Centre for Marine Research (HCMR) and cultures of *Tetraselmis* sp. (Florida Aqua Farms - 352-567-0226) and *Heterosigma akashiwo* (CCAP 934/7, Oban, Scotland) were maintained in the algal collection of aquatic ecology laboratory of University of Glasgow. All cultures were incubated at 21 °C, in a 24-hour continuous photoperiod under fluorescent light and in artificial ultra-pure autoclaved seawater with salinity 35 psu. Mother cultures (2 L) had a continuous ventilation system whereas in treatment and control cultures (200 mL), regular stirring was applied instead.

All cultures were initiated with a concentration of 5000 cells mL^−1^ apart from *Phaeocystis* sp. which was inoculated with 10,000 cells mL^−1^ due to the much higher population carrying capacity of this species. This was to achieve a synchronization among treatment cultures and MCs from which they received filtrate. Synchronization refers to the growth stage of the cultures, i.e. all cultures being at the stationary stage during filtrate additions. MCs and treatments, including controls, were cultured in medium F/2 Guillard (1975) (see supplementary material) but with P-limitation (i.e. 3 μΜ instead of 36μΜ P) because allelopathy is known to be higher under nutrient-limited conditions [17], [27], [35], [46], [47]. In this study we focus on P-limitation, because previous research from coastal ecosystems, where these species are usually encountered, has shown that P limitation can occur on a seasonal basis depending on the intensity of freshwater terrestrial inflows into the coastal environment [48], [49]. All species were acclimated to the experimental conditions for at least a month prior to the initiation of the experiment. These conditions (including light and growth medium) were the same across all species, as we aimed to test for species interactions under conditions when species co-occur in nature.

### 2.3 Sampling & metabolomics analysis

At each of the two sampling points (pre-filtrate addition and post-filtrate addition), 5 mL were removed from each culture for cell counting, they were preserved with a drop of Lugol solution and stored in the refrigerator (4 °C). Cell counting was carried out under an optical Leica microscope at 200× magnification using Fast-Read® 102 disposable counting chambers (immune systems). An additional 50 mL aliquot of each culture was collected and from this 25 mL was filtered onto 25 mm Whatman GF/C glass microfiber filters for chlorophyll-a (Chla) analysis and the other 25 mL was filtered for intracellular P analysis, while the 50 mL filtrate was used to evaluate the residual P in the medium. Filters for Chla and intracellular P analysis were placed in aluminum foil and together with samples for nutrients (PO_4_) were frozen at −20 °C. Chla and residual phosphorus in the medium were quantified according to Parsons et al. (1984) (see supplementary material). Determination of intracellular P was carried out according to Caceres et al. (2019) and details are provided in the supplementary material under “Methods”.

Metabolomics analysis was carried out to detect the unique exometabolites released by each species in the medium. These were determined from 5 mL samples taken from our control cultures of the four species at the second sampling point. Samples for metabolomics analysis (5 mL) were quenched by rapidly cooling cells in ice for 10 min. The cells were removed by centrifugation for 10 min at 3000*g* at 4 °C and 25 μL of supernatant was taken from all the samples. In each sample 1 mL of chloroform/methanol/water (1:3:1 v/v/v) was added, the samples were vortexed for 1 min and centrifuged again for 3 min at 10,000*g* at 4 °C. Finally, 300 μL of supernatant was added in cryovials (three times from each sample to create back-up technical replicates) and stored at −80 °C. A sample from the growth medium was also taken (i.e. artificial saltwater with F/2, P/24 nutrients) to control for any substances present therein. For quality analysis purposes, a pooled sample of all the samples at each sampling time point was also used for a metabolomics analysis. An untargeted metabolomics approach was employed to determine the metabolic profiles of the cultures. Liquid chromatography-mass spectrometry (LC-MS) analysis was performed with a Thermo Orbitrap Q-Exactive mass spectrometer interfaced to a Dionex UltiMate 3000 RSLC system. Samples (10 μL) were injected onto a Merck Sequant ZIC-pHILIC column (150 mm × 4.6 mm; 5 μm) maintained at 30 °C. Mobile phase A consisted of water containing 20 mM ammonium carbonate and mobile phase B consisted of acetonitrile. The initial conditions for analysis were 20% mobile phase A–80% mobile phase B and the percentage of mobile phase A was increased to 95% over 15 min with a hold for 2 min before re-equilibration to the starting conditions over 9 min. The flow rate was 0.3 mL/min. Analysis was operated in polarity switching mode over the mass-to-charge ratio (*m*/*z*) range of 70 to 1050 at a resolution of 70,000. Data sets were processed with IDEOM [50] which uses the XCMS [51] and mzMatch [52] software in the R environment and PiMP [53]. The levels of reliability of the spectral assignment to metabolites, as defined by the Metabolomics Standard Initiative were as follows: ‘MSI:1 (identified metabolites)’: high resolution mass (3 ppm) and retention time (5%) matched to an authentic standard, ‘MSI:2 (putatively annotated compounds)’: high resolution mass matched to a public library (3 ppm).

### 2.4 Data analysis

For each of our response variables (cell counts, Chla, medium PO_4_ and intracellular P) the effect of the addition of exometabolites of foreign species on each target species was tested using the following statistical approach. We first fitted a model for each of our four response variables with which we accounted for any differences -in the response variable- between the cultures of a given species prior to the filtrate additions from the “foreign” species. An example of the Generalized Linear Model (GLM) structure in the case of Chla was:

$$\mathrm{Chl}a_{\mathrm{post}-\mathrm{addition}}=\mathrm{Chl}a_{\mathrm{pre}-\mathrm{addition}}+\left[\begin{array}{c} f_{\mathrm{Tet}}\\ f_{\mathrm{Het}}\\ f_{\mathrm{Pha}}\\ f_{\mathrm{Tha}} \end{array}\right]\times \left[\begin{array}{c} C_{\mathrm{Tet}}\\ C_{\mathrm{Het}}\\ C_{\mathrm{Pha}}\\ C_{\mathrm{Tha}} \end{array}\right]$$

where, *f*_species_ stands for the filtrate addition level, *C*_species_ stands for the species' culture and their product is testing for the interaction between the two factor variables. After fitting the models, we extracted the fitted values for each response variable and used these instead of the original data values for further analysis. This approach is helping us isolate differences between cultures prior to filtrate addition from the actual treatment effects. Using the fitted values for each response variable we then carried pairwise comparisons between treatment cultures and their respective controls based on the Tukey method for adjustment of means using the package emmeans. The outcome of this analysis was a “contrast value” indicating the difference between treatment and respective control and a corresponding *p*-value. This difference between treatment and control (from now on referred to as Δ) indicated a significant positive effect of exometabolites on the tested variable when Δ >0 and *p* < 0.05, a significant negative effect when Δ < 0 and *p* < 0.05, and no effect when *p* > 0.05. To illustrate, when the target species *Heterosigma* (Het) receives filtrate from *Tetraselmis* (f_Tet_), then the effect on Chla would be negative if ΔChla<0, *p* < 0.05, indicating that Chla in the replicates of the Het cultures treated with “foreign” species filtrate was on average significantly lower than the replicates of the control Het which received plain artificial seawater.

To determine exometabolites that were excreted by the species, we first excluded from our analysis all the metabolites that were present in a sample taken from the growth medium. Then we fitted the following model, with the response variable being each metabolite and the explanatory variable being the factor variable “species” with four levels corresponding to the control cultures of each species:

$$\mathrm{Metabolite abundance}=\left[\begin{array}{c} {\mathrm{Control}}_{\mathrm{Tet}}\\ {\mathrm{Control}}_{\mathrm{Het}}\\ {\mathrm{Control}}_{\mathrm{Pha}}\\ {\mathrm{Control}}_{\mathrm{Tha}} \end{array}\right]$$

Using the F-ratio and associated *p*-value from this analysis, we identified exometabolites that showed significant differences between the 4 species. These were further explored with pairwise comparisons using the package emmeans for pairwise comparison of means based on the Tukey method. Metabolites that had significantly higher abundance in specific species were discussed as potential substances driving the observed responses in target species at the physiological level (i.e. biomass production, nutrient use).

Statistics were performed using R programming language version 4.0.5 (13-04-2021) [54] in the software R-Studio Desktop. Packages ggplot2 [55], and ggpubr [56] was used for data visualization and emmeans (i.e. Estimated Marginal Means (Least-Squares Means)) was used for pairwise comparison of means based on the Tukey method [57]. Function glm in package stats [52] was used for the GLM.

## 3 Results

### 3.1 Response of target microalgae to filtrate addition of “foreign” microalgae

**Fig. 2:**
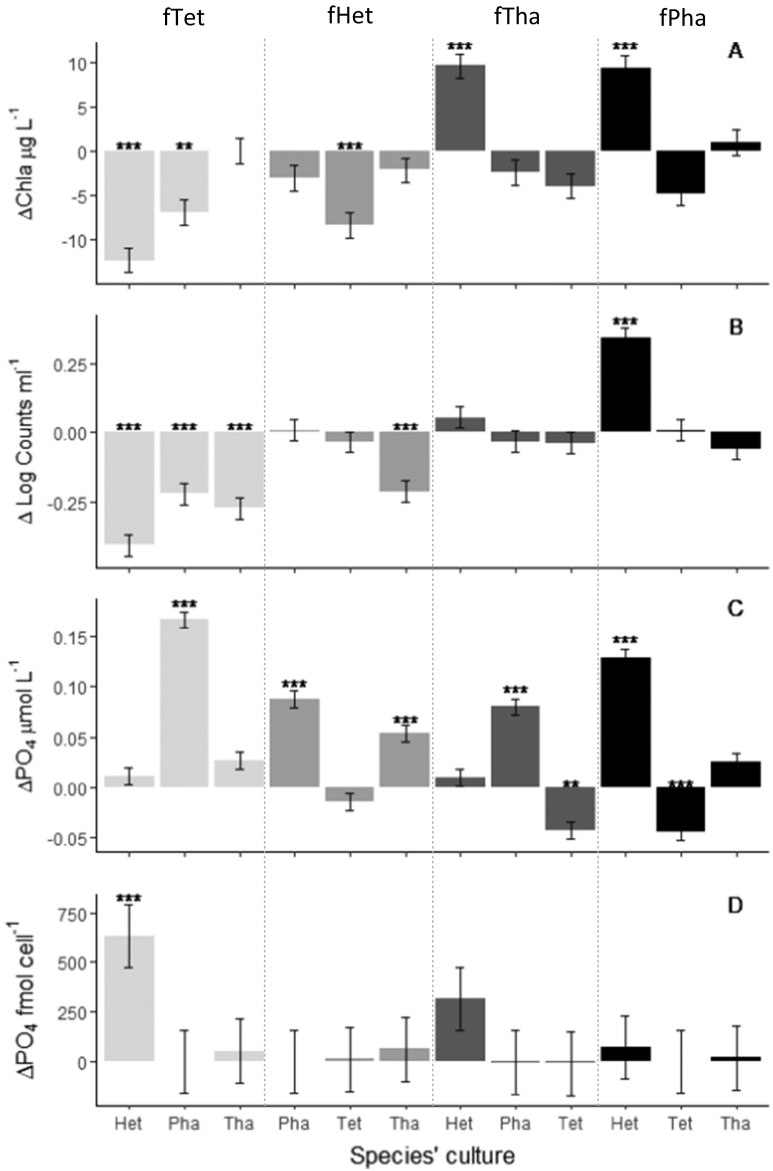
Effect of filtrate with exometabolites from foreign microalgae species on biomass production and resource use of target microalgae species: biomass production expressed as Chla (panel A) and log cell number (panel B), and resource use of limiting nutrient P expressed as residual PO4 in the medium (panel C) and intracellular P (panel D). For each of the four response variables examined, Δ indicates the difference between treatment and the respective control which was the culture of that species treated with plain medium. The x-axis presents the 12 cultures across our 4 species (*Heterosigma*-Het, *Phaeocystis*-Pha, *Thalassiosira*-Tha, *Tetraselmis*-Tet) that were being treated with filtrate from the MCs of the “foreign” species (f_Tet_, f_Het_, f_Tha_, f_Pha_). Annotations of significance levels based on Tukey adjusted pairwise comparisons: *p* > 0.05, *: *p* ≤ 0.05, **: *p* ≤ 0.01, ***: *p* ≤ 0.001.

Our results show that filtrate from *Heterosigma* (fHet) and *Tetraselmis* (fTet) can cause a significant biomass suppression in other species (expressed either as photosynthetic biomass or counts (ΔChla < 0 & ΔCounts < 0, *t*-test, *p* < 0.05) (Fig. 2A & B)). Only exception to this was *Phaeocystis* which remained unaffected by the filtrate addition of *Heterosigma* (fHet). On the other hand, cultures treated with filtrate from *Thalassiosira* (fTha) and *Phaeocystis* (fPha), showed biomass enhancement in the species *Heterosigma* compared to the respective controls (ΔChla > 0 & ΔCounts > 0, *t*-test, *p* < 0.05) (Fig. 2A&B).

Our results also show that residual PO_4_ measured in the treated culture medium was increased compared to the controls in some treatments, including all *Phaeocystis* cultures, suggesting it remained unused (ΔPO_4_ > 0, *t*-test, *p* < 0.05), was negative in two *Tetraselmis* treatments suggesting that more was used compared to controls (ΔPO_4_ < 0, *t*-test, p < 0.05), and remained unaffected in another 5 treatments (ΔPO_4_ = 0, *t*-test, *p* > 0.05) (Fig. 2C). Regarding intracellular P concentrations, no differences were observed between treatments and controls (ΔPO_4_ = 0, *t*-test, *p* > 0.05) with the exception of *Heterosigma* treated with *Tetraselmis* filtrate (fTet) whereby although the residual PO_4_ in the medium remained unchanged, there was a significant increase in intracellular PO_4_ (Fig. 2D).

### 3.2 Exometabolites measured in the filtrate of “foreign” microalgae

**Fig. 3:**
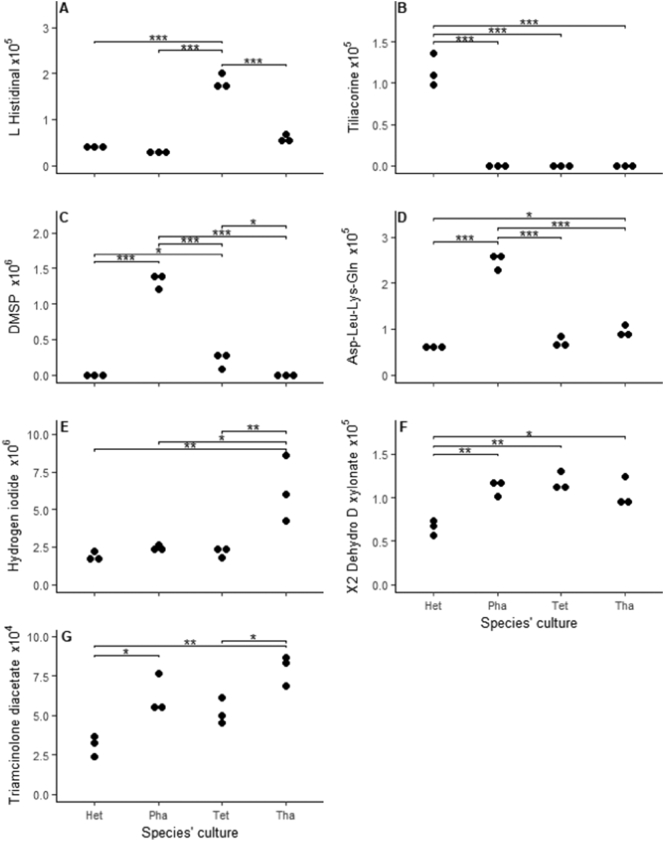
Putative exometabolites measured from the filtrate of the control monocultures of the four microalgae species (*Heterosigma*-Het, *Phaeocystis*-Pha, *Thalassiosira*-Tha, *Tetraselmis*-Tet) that were either significantly higher either in specific species or across subgroups of the four microalgae species (GLM, *p* < 0.05). Annotations of significance levels based on Tukey adjusted pairwise comparisons: *p* > 0.05, *: *p* ≤ 0.05, **: *p* ≤ 0.01, ***: *p* ≤ 0.001.

58 detected ion signals showed statistically significant differences between the four microalgae species (GLM, *p* < 0.05). From these, 23 were selected as being present in the cultures and not in the growth medium (GM), indicating they were produced endogenously by the algal species. From those, a metabolite putatively identified as L-histidinal was significantly elevated in cultures of *Tetraselmis* (Fig. 3A). Further, metabolites putatively annotated as tiliacorine and hydrogen iodide were present in higher levels in *Heterosigma* and *Thalassiosira* cultures respectively (Fig. 3B & E), together with several unidentified ion signals (SFig. 1A, B & G). The haptophyte *Phaeocystis* was found to have the largest number of significantly elevated exometabolites. Specifically, cultures from this species showed increased concentrations of a metabolite putatively annotated as *S*,*S*-dimethyl-beta-propiothetin also known as dimethylsulfoniopropionate (DMSP), a tetra peptide Asp-Leu-Lys-Gln (Fig. 3C & D) as well as four uncharacterized ion signals (SFig. 1C–F).

## 4 Discussion

**Fig. 4:**
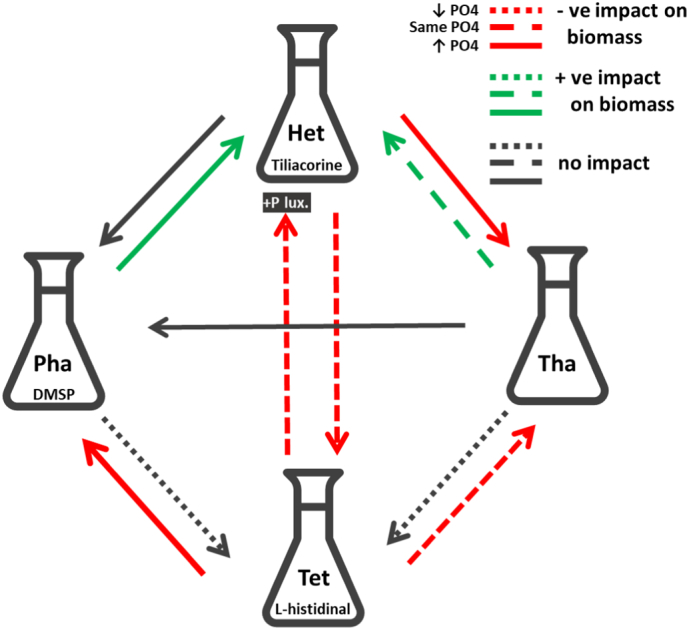
Range of responses of “target” microalgae to exometabolites of “foreign” microalgae (*Heterosigma*-Het, *Phaeocystis*-Pha, *Thalassiosira*-Tha, *Tetraselmis*-Tet). Our crossed experimental design enabled the testing of directional effects of all possible combinations of four species belonging to different taxonomic groups and representing HAB and non-HAB species: Het-Ochrophyta and Pha-Haptophyta (HAB), Tet-Chlorophyta and Tha-Bacillariophyta (non-HAB).

Here we investigate responses of “target” microalgae to exometabolites of “foreign” microalgae by testing for directional effects of all possible combinations of four species belonging to different taxonomic groups and representing HAB and non-HAB species (Fig. 4). Our experiment enabled to disentangle the effect of chemical interference from that of nutrient competition by using cell-fee culture medium from the “foreign” species' cultures. Our findings show that responses of target species strongly depend on which foreign species is affecting them. They also reveal that chemical interference through exometabolites of foreign species can cause a range of responses to target microalgae the most common being biomass suppression (e.g. all species under the influence of *Tetraselmis*), and less common being biomass enhancement (e.g. *Heterosigma* under the influence of *Phaeocystis*) (Fig. 4). The increased intracellular P content in *Heterosigma* cells under the influence of *Tetraselmis* also demonstrates the potential of microalgae for growth strategy shifts as a response to sensing competitors. Indeed, although this strategy has been previously documented in *Heterosigma* which was actively migrating to accumulate P from nutrient-rich deep water layers [8], [58] it has not been previously documented as a response to sensing of competitor species. These findings have obvious implications in the way we understand ecological interactions between microalgae, indicating that interspecific interactions extend beyond nutrient competition and allelopathy and should be carefully factored in species population models predicting assemblage species composition, biodiversity or biomass.

Our findings have also important implications for industrial production of microalgae as they are challenging the view that co-cultures can be used for maximizing algal biomass in bioreactors [59], [60]. Specifically, here we show that the most common effect of foreign species' filtrate is a decrease in the biomass of the target species and this response was observed for all 3 species under the influence of *Tetraselmis* and 2 species under the influence of *Heterosigma*. This is important as *Tetraselmis* is well known for its commercial potential for biofuel production and high value products [61], [62], [63] as well as fish and shellfish aquaculture feed [64] due to its high-lipid content strain [65]. In co-culture settings within bioreactors, such negative effects due to chemical interference can only be exacerbated due to nutrient competition thus leading to an undesirable underyielding relative to the respective monocultures. Although underyielding was in fact observed in previous experimental studies using species co-cultures [32], [66] the effect of chemical interference was not disentangled from nutrient competition obscuring the mechanisms behind the observed yield patterns.

Interestingly, the species that was shown to produce the most negative responses to target species was *Tetraselmis* sp., a species not considered as harmful. Indeed, our findings suggest that species traditionally thought of as non-HAB, might be capable of causing biomass inhibition to other microalgae species. The absence of a known toxin in this case, raised the question whether the response of target species is due to sensing and reacting to exometabolites of *Tetraselmis* via e.g. a growth strategy shift or rather are directly affected by exometabolites that have algicidal action. Further insights to this were obtained by our comparative metabolomics analysis which showed that L-histidinal, a biosynthetic precursor of the amino acid L-histidine, was the only exometabolite present in significantly higher abundance in *Tetraselmis*. A previous study found that L-histidine produced by bacterial cultures of *Bacillus* sp. strain B1 could have acted as an algicidal of a *Phaeocystis globosa* HAB in Zhuhai, China [67], [68]. This suggestion was confirmed by further experimental work testing the effects of commercially purchased L-histidine on *Phaeocystis globosa* cultures [69]. Our study indicates that L-histidinal can in fact also originate from microalgae species and could adversely affect biomass production of species of different taxonomic groups (Bacillariophyta, Haptophyta, Ochrophyta). Therefore, the potential of this compound as a novel biotoxin used to regulate natural HABs, should be further explored.

*Heterosigma akashiwo* also caused negative effects on growth of 2 other target species. This HAB raphidophyte species is known to cause extensive fish-killing blooms worldwide [43]. The ichthyotoxicity of *H. akashiwo* is still under investigation although a number of potential mechanisms have been proposed, such as the production of Reactive Oxygen Species (ROS) compounds, neurotoxins, hemolytic compounds etc. The bloom success of *H. akashiwo* has been associated with its production of allelochemicals capable of inhibiting the growth of co-occurring microalgae, e.g. high-molecular-weight polysaccharide-protein complexes (Wang et al. [70] and references therein). A putative metabolite uniquely identified in *H. akashiwo* for the first time in our study is Tiliacorine, an alkaloid originally identified in the edible plant *Tiliacora triandra* with pharmaceutical potential due to confirmed cytotoxicity of the malarian inducing protozoan *Plasmodium falciparum* [71] and the tuberculosis inducing bacterium *Mycobacterium tuberculosis* [72]. Our study indicates the potential of this substance as an algicidal agent and merits further experimentations.

Positive effects on both cell counts and photosynthetic biomass were observed only in the species *Heterosigma* under the influence of *Phaeocystis* sp. *Phaeocystis* showed significantly increased metabolite signals of the putatively annotated as *S*,*S*-Dimethyl-beta-propiothetin, also known as dimethylsulfoniopropionate (DMSP). DMSP is actively synthesized by certain microalgae, where it is thought to have osmotic, cryoprotective, predator deterring and antioxidant properties [73] and is produced in very high concentrations during microalgal blooms in the field [74]. Previous studies have found that this might be an important source of carbon and sulfur for bacteria in aquatic systems [30], [75], [76]. The fact that *Heterosigma* showed increased yield while at the same time leaving the limiting phosphate unused in the medium, suggests that this known mixotroph might have switched the growth strategy from autotrophy to heterotrophy. The latter could have been triggered by boosted bacteria growth due to DMSP in the *Phaeocystis* treatments. Enhanced microalgal growth due to facilitation effects from bacteria as well as the role of DMSP in these mutualistic associations is being studied [77]. Furthermore, the potential of algae-bacteria interactions, also mediated by DMSP is being increasingly explored in the field of biofuels [78]. The indirect link between a microalgal exometabolite boosting mixotrophic algal yield through bacteria growth merits further research due to the important potential of maximizing algal biomass without nutrient supply, as already highlighted in previous studies for nitrogen [79].

The present study highlights the importance of exometabolites in microalgae interactions and the complexity of responses they invoke. Growth facilitation, growth inhibition, algicidal action, strategies shifts, all seem possible responses to foreign species exometabolites, irrespective of known toxicity effects of the studied species. Furthermore, metabolites that mediate the above interactions could not be assigned to a putative metabolite, highlighting an important gap in metabolite research. Although beyond the scope of the present study, some of our findings suggest the importance of algae-bacteria associations, especially in the case of mixotroph species. Our findings, together with the recognized gaps in our knowledge of microalgae metabolites, have important implications in plankton succession prediction modeling and applied microalgae research, including biofuel industry and water remediation.

## CRediT authorship contribution statement

NGA: Investigation, Methodology, Formal analysis, Visualization, Writing - Original Draft.

ES: Conceptualization, Methodology, Formal analysis, Visualization, Funding acquisition, Writing - Review & Editing.

ML: Investigation, Writing - Review & Editing.

IV: Methodology, Resources, Writing - Review & Editing.

PW: Methodology, Investigation, Writing - Review & Editing.

CR: Methodology, Investigation, Writing - Review & Editing.

SS: Conceptualization, Methodology, Formal analysis, Visualization, Supervision, Resources, Funding acquisition, Writing - Review & Editing.

## Declaration of competing interest

The authors declare that they have no known competing financial interests or personal relationships that could have appeared to influence the work reported in this paper.

## Appendix A Supplementary data

Supplementary material including the method for growth rate estimation for our cultures, the medium used, the Chla and residual P references, the method for intracellular P estimation and the results of the metabolites that were not reported in the main text. Image 1
