# Supplementary material for: Microalgae show a range of responses to exometabolites of foreign species
Source: Algal Res. 2022 Mar;62:None. doi: 10.1016/j.algal.2021.102627 (PMC8924005; doi:10.1016/j.algal.2021.102627)
Supplement: Supplementary file 1 — Supplementary material including the method for growth rate estimation for our cultures, the medium used, the Chla and residual P references, the method for intracellular P estimation and the results of the metabolites that were not reported in the main text. [file mmc1.docx]

***Supplementary material for Apostolopoulou et al***

***METHODS***

***Determination of growth of different species’ cultures***

A pre-experimental monitoring of microalgae’s growth rates occurred in HCMR in order to gain an understanding of the capability of selected microalgae strains to grow under P limitation (i.e. F/2 medium with P/24). Before the experiment started we had monitored growth rate of pilot cultures by measuring cell concentration according to Eq. (2) as a function of time to define the appropriate time to start the experiment. It was noticed that *Thalassiosira* sp. and *Phaeocystis* sp. lagged four days behind the other species. Experimental MCs and treatments were initiated at a different time points to ensure that they would all reach stationary phase simultaneously.

*Growth rate (d^-1^)= ln(N_2_/N_1_) (2)*

*(t_2_-t_1_)*

Where N1 was the cell concentration (ml^-1^) at time (i.e. in days) t1; at this point the culture had passed the lag phase and N2 was the cell concentration at time t2; when culture had reached its maximum cell concentration.

***Determination of Intracellular P***

Determination of intracellular P was carried out according to Caceres et al (2019). For the digestion of organic phosphorus, the filters were placed in glass vials with 2 ml of concentrated (70%) nitric acid. Glass loose caps were placed on each vial and they were deposited on a hotplate at a gentle boil (i.e. ~121^o^C) for one hour. When the samples cooled down the filters were rinsed four times with 10ml of ultra-pure water. The 10 ml of aliquots were transferred carefully to a volumetric flask and flasks were filled up to a total volume of 70 ml with ultra-pure water. Afterwards, the solution was neutralized (ie pH=7) by adding 5ml 5M sodium hydroxide and 2 drops of p-nitrophenol indicator (0.1% solution). Further drops of 5M sodium hydroxide were added until the indicator turned yellow. Thereafter, 0.5M sulphuric acid were added dropwise until the indicator turned colorless. Intracellular P were quantified following the same procedure as for the residual P in the medium (ie see above). Finally, P quota (µmol cell^-1^) was estimated from the inorganic P concentration measured in the digested solution according to the following equation (Eq. 1):

*Q= (P_ds_ – P_ds_blank_)*Vol_flask_ (1)*

*N*Vol_f_*

Where:

*P_ds_*= Digested solution, *Vol_flask_* = Volume of the volumetric flask (V=100 ml) in L, *N* = Cell concentration, cells/L, *Vol_f_* = Volume of culture filtered (V=25 ml) in L, *P_ds_blank_* = Phosphorus concentration measured in the digested solution for the blank

***Culture medium***

All of our species were cultured in medium F/2 Guillard (1975)

Guillard, RRL 1975. Culture of phytoplankton for feeding marine invertebrates. pp 26-60. In Smith, W.L. and Chanley, M.H. (Eds) Culture of Marine Invertebrate Animals, Plenum Press, New York.

***Chla and residual phosphorus analysis***

Chla and residual phosphorus in the medium were quantified according to Parsons et al. (1984).

Parsons TR, Maita Y, Lalli CM (1984) A manual of chemical and biological methods for seawater analysis. Pergamon Press, Oxford

**RESULTS**


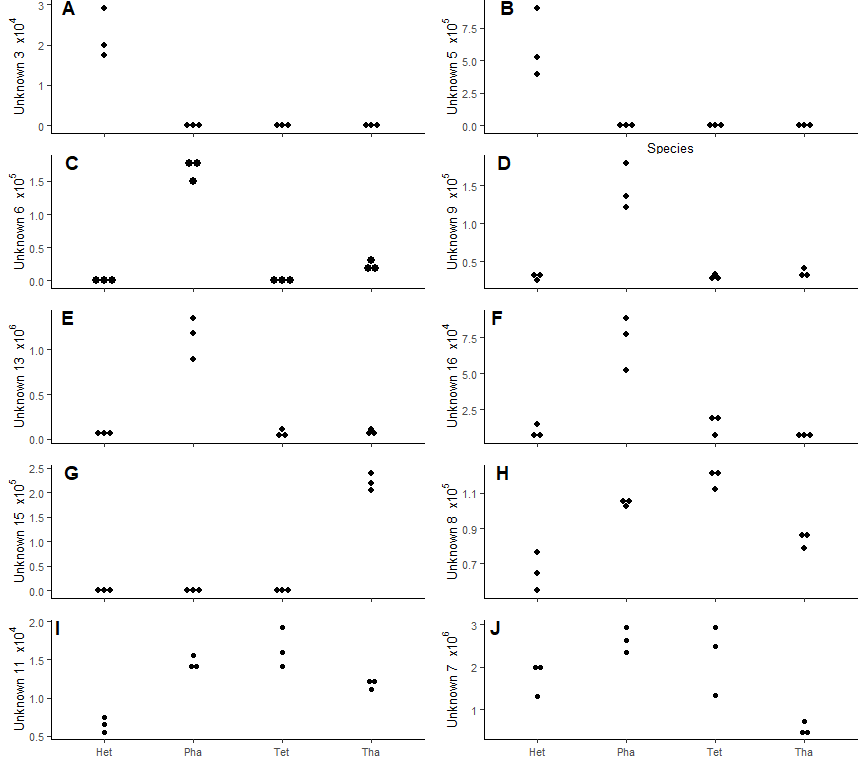


Figure S1. Unknown metabolites for which significant differences were detected between the four experimental species (Hetrosigma-Het, Phaeocystics -Pha, Tetraselmis-Tet,Thalassiosira -Tha) and were not detected in the growth medium suggesting that they were endogenously produced by the species. PanelsA-B indicate 2 metabolites present in abundance only in Heterosigma, Panels C-F indicate 4 metabolites present in abundance only in Phaeocystis, panel G indicates one metabolite abundant in Thalassiosira, whereas panels H-J indicate metabolites shared across three of the species.
